# Immunomodulation for Severe COVID-19 Pneumonia: The State of the Art

**DOI:** 10.3389/fimmu.2020.577442

**Published:** 2020-11-09

**Authors:** Yinhua Zhang, Yuanyuan Chen, Zhongji Meng

**Affiliations:** ^1^ Department of Infectious Diseases, Taihe Hospital, Hubei University of Medicine, Shiyan, China; ^2^ Hubei Clinical Research Center for Precise Diagnosis and Treatment of Liver Cancer, Taihe Hospital, Hubei University of Medicine, Shiyan, China; ^3^ Institute of Biomedical Research, Taihe Hospital, Hubei University of Medicine, Shiyan, China; ^4^ Hubei Key Laboratory of Embryonic Stem Cell Research, Taihe Hospital, Hubei University of Medicine, Shiyan, China

**Keywords:** coronavirus disease 2019, severe acute respiratory syndrome coronavirus 2, pneumonia, critical illness, immunomodulation

## Abstract

COVID-19 has become a worldwide pandemic caused by the novel coronavirus named severe acute respiratory syndrome coronavirus 2 (SARS-CoV-2). Severe cases of COVID-19 have accounted for 10–20% of all infections, leading to more than 500,000 deaths. Increasing evidence has suggested that the inflammatory cytokine storm originating from the anti-SARS-CoV-2 immune response plays an important role in the pathogenesis of critically ill patients with COVID-19, which leads to mixed antagonistic response syndrome (MARS). In the early stage of severe COVID-19, systemic inflammatory response syndrome causes acute respiratory distress syndrome, multiple organ dysfunction syndrome, and even multiple organ failure. In the late stage of severe disease, increased production of anti-inflammatory cytokines drives the immune response to become dominated by compensatory anti-inflammatory response syndrome, which leads to immune exhaustion and susceptibility to secondary infections. Therefore, precise immunomodulation will be beneficial for patients with severe COVID-19, and immunosuppressive or immune enhancement therapy will depend on the disease course and immune status. This review summarizes the current understanding of the immunopathogenesis of severe COVID-19, especially the role of the inflammatory cytokine storm in disease progression. Immune indicators and immunotherapy strategies for severe COVID-19 are reviewed and the potential implications discussed.

## Introduction

Coronavirus disease 2019 (COVID-19) is spreading rapidly worldwide. As of June 19, 2020, more than 8 million confirmed cases have been reported in more than 200 countries (1), and the number of cases continues to increase rapidly. On February 11, 2020, based on gene sequence homology, the Coronaviridae Study Group of the International Committee on Taxonomy of Viruses concluded that the new coronavirus is a sister virus of severe acute respiratory syndrome coronavirus (SARS-CoV) (2), which is also a member of the *Betacoronavirus* genus, and officially named it SARS-CoV-2. At the same time, the World Health Organization renamed “novel coronavirus pneumonia” to “COVID-19.” In general, coronaviruses infecting humans can be divided into low-pathogenic human coronaviruses (HCoVs), including HCoV-229E, HCoV-OC43, HCoV-NL63, and HCoV-HKU, and highly pathogenic coronaviruses, such as SARS-CoV and Middle East respiratory syndrome coronavirus (MERS-CoV) (3). Low-pathogenic HCoVs infect the upper respiratory tract and cause seasonal mild to moderate cold-like respiratory diseases in healthy people. In contrast, highly pathogenic HCoVs infect the lower respiratory tract, causing severe pneumonia, which can lead to fatal acute lung injury (ALI) and acute respiratory distress syndrome (ARDS), resulting in high morbidity and mortality (4, 5). At the time of this writing, the incidence of ARDS in COVID-19 patients has been 14.8%, severe cases have accounted for 18.1% of all infections, and the number of deaths has been rising (6). Immune dysregulation induced by highly pathogenic HCoVs, characterized by a large amount of inflammatory cell infiltration and the production of large amounts of proinflammatory cytokines/chemokines, leading to systemic inflammatory response syndrome (SIRS) and ARDS, plays a critical pathogenic role in disease deterioration and even leads to the death of patients. In this review, we focus on immunological assessment and immunotherapy for patients with severe COVID-19 pneumonia.

## Immunopathogenesis of COVID-19 Pneumonia

SARS-CoV-2 is homologous to SARS-CoV at both the nucleotide and amino acid levels (2). Findings from immunohistochemical and *in situ* hybridization analyses on autopsied patients with SARS-CoV pneumonia (hereinafter referred to as SARS) confirmed that the SARS-CoV spike protein is expressed only in angiotensin-converting enzyme II (ACE2)^+^ cells (7). Moreover, SARS-CoV-2 enters cells *via* ACE2 receptors (8). It is speculated that SARS-CoV-2 may share similar pathogenic mechanisms and pathological changes with SARS-CoV. Compared with the SARS-CoV receptor binding domain (RBD), the SARS-CoV-2 RBD exhibits a tighter conformation in its human ACE2 (hACE2) binding ridge; moreover, several residue changes in the SARS-CoV-2 RBD stabilize the two binding hot spots in the RBD/hACE2 interface (9). These structural features of the SARS-CoV-2 RBD enhance its binding affinity for hACE2, suggesting that SARS-CoV-2 is more likely than SARS-CoV to infect humans (10).

COVID-19 patients can be clinically characterized into two types: those with mild cases, which can be quickly controlled or self-limiting, and those with severe cases, which often progress to severe or critical illness and can be life-threatening. Clinical manifestations suggest that old age, the presence of comorbidities, a high respiratory viral load, and detection of viral RNA in the blood are high-risk factors for severe disease. A prospective study demonstrated that the clinical course of severe SARS has three unique stages. In the first stage, patients experience fever, coughing, and other symptoms, and the fever then improves. In the second stage (after an average of 8–9 days), patients again develop high fever, accompanied by hypoxemia and pneumonia-like symptoms. In the third stage, patients progress to ARDS, which is life-threatening. Plots of the viral load in the respiratory tract exhibit an inverted V-shape throughout the course of the disease, with the viral load peaking on day 10. The continued deterioration of the disease in the later stage is considered to be unrelated to viral replication; rather, it is suspected to be associated with the immune response.

The pathological changes in SARS have been summarized as lung lesions, immune organ damage, systemic vasculitis, and changes in systemic toxicity (11). Pulmonary lesions include extensive bilateral consolidation; localized hemorrhagic necrosis; desquamative alveolitis; bronchitis; alveolar epithelial cell hyperplasia and desquamation; exudation of alveolar proteins, monocytes, lymphocytes, plasma cells, and alveolar epithelial cells; formation of an inner hyaline membrane; and the presence of viral inclusion bodies. Massive necrosis of splenic lymphatic tissue and local necrosis of lymph nodes are observed. Systemic vasculitis includes interstitial edema in the heart, lungs, liver, kidneys, adrenal glands, and striated muscles; localized fibrinoid necrosis; and infiltration of monocytes, lymphocytes, and plasma cells into the vascular wall. The small veins exhibit thrombosis. Changes in systemic toxicity include degeneration and necrosis of parenchymal cells in the lungs, liver, kidneys, heart, and adrenal glands. The lungs, immune organs, and systemic small blood vessels are believed to be the main targets of viral attack. Extensive lung consolidation, diffuse alveolar injury and hyaline membrane formation, respiratory distress, and decreased immune function are the main causes of death. Autopsy of another eight patients with SARS confirmed the detection of SARS-CoV particles and genomic sequences in many circulating lymphocytes, monocytes, and lymphoid tissues. In addition, SARS-CoV particles and genomic sequences have been detected in respiratory epithelial cells, intestinal mucosa, distal renal tubular epithelial cells, brain neurons, and macrophages in various organs. Therefore, the comprehensive pathogenesis of SARS is characterized by aberrant immune responses and lung injury. ACE2 expression in humans has been detected *via* immunohistochemistry in 15 different organs and tissues across 93 patients (oral and nasal mucosa, nasopharynx, lungs, stomach, small intestine, colon, skin, lymph nodes, thymus, bone marrow, spleen, liver, kidneys, and brain) (12). ACE2 proteins are most significantly expressed on the surface of alveolar epithelial cells and small intestinal epithelial cells. In addition, ACE2 was present on arterial and venous endothelial cells and arterial smooth muscle cells of all organs studied. ACE2 has also been detected in monocytes/macrophages (13) and in CD68^+^CD169^+^ macrophages in the spleens and lymph nodes of patients with COVID-19 (14). TMPRSS2 has been identified as an alternative receptor for SARS-COV-2 and has been found on lymphocytes and macrophages (15). Furthermore, SARS-COV-2 viral particles or proteins have been found in the spleens and hilar lymph nodes of patients who died of COVID-19 (16). These results suggest that immune cells/organs may be attacked by SARS-COV-2 infection in addition to the systemic effect of the abnormal immune response to the virus. The results from 4 SARS patients showed high expression levels of proinflammatory cytokines in SARS-COV-infected ACE2^+^ cells but not in uninfected ACE2^+^ cells (7), suggesting that proinflammatory cytokines play an important role in disease development. These results are consistent with the increase in neutrophils and monocytes and the decrease in CD4 and CD8 T cells in the peripheral blood of patients with fatal SARS.

Similar pathological changes have been found in the lungs of COVID-19 patients. Pathological examination in patients who died of COVID-19 showed extensive lung consolidation, diffuse alveolar injury, robust macrophage infiltration, alveolar hyaline membrane formation, and respiratory distress (17). A large amount of clinical data has shown that COVID-19 patients exhibit significantly reduced peripheral blood lymphocyte counts, which is associated with disease severity, suggesting a key role of this parameter in predicting severe COVID-19 disease (10, 18). The early decrease in peripheral blood lymphocytes may be attributed to changes in lymphocyte distribution due to their infiltration into the lungs to participate in antiviral inflammatory responses. However, late in the disease course, the peripheral blood lymphocyte count in patients with severe COVID-19 continues to decline, and atrophy of lymphatic organs and reductions in bone marrow hyperplasia have been found in patients who died of COVID-19. How do these effects arise? Do they occur due to an excessive anti-inflammatory response, or does SARS-CoV-2 directly infect lymphocytes and cause immune cell destruction? ACE2 expression has not been detected in immune cells or organs (12), and no evidence indicates that SARS-CoV-2 infects immune cells *via* ACE2.

## The Role of the Inflammatory Cytokine Storm in Disease Progression in Critically Ill Patients With COVID-19

The inflammatory cytokine storm, also known as cytokine storm or cytokine release syndrome (CRS), is a severe excessive immune response caused by a positive feedback loop between cytokines and immune cells. The symptoms of CRS include high fever, erythema, edema, extreme fatigue, and nausea, and it is an important cause of sepsis and multiple organ failure (19).

The innate immune response is the first line of defense against viral infections. Cytokines and chemokines play important roles in the immune response and immunopathology of viral infections. Dysregulated, excessive immune responses lead to SARS immunopathology (20). Macrophages are the key sentinel cells in the respiratory system. The poor ability of SARS-CoV-infected macrophages to produce chemokines and interferon β (IFN-β), a key component of innate immunity, may be an early pathogenic mechanism of SARS (21). In vitro assays have demonstrated that dendritic cells (DCs) infected with SARS-CoV produce low levels of the antiviral cytokines IFN-α and IFN-β; moderate levels of the proinflammatory cytokines tumor necrosis factor (TNF) and interleukin-6 (IL-6); and high levels of the inflammatory chemokines CCL3, CCL5, CCL2, and CXCL10 (22). Another *in vitro* study showed that, upon infection with SARS-CoV, alveolar epithelial cells (A549) produce large amounts of CCL3, CCL5, CCL2, and CXCL10, while mononuclear cells (THP-1) produce large amounts of CCL2, CXCL8/IL-8, CCL3, CXCL10, CCL4, and CCL5 (23). These cytokines/chemokines are the key factors for the chemotaxis of neutrophils, monocytes, and activated T cells, and their excessive production may lead to dysregulation of the immune response to SARS-CoV infection. These studies showed that the dysregulation, delay, and/or exaggeration of the responses of SARS-CoV-infected macrophages, DCs, and alveolar epithelial cells to cytokines and chemokines may play an important role in the deterioration of patients with SARS.

Young BALB/c mice infected with the MA15 virus, a type of SARS-CoV to which mice are susceptible, exhibit the pathological features of diffuse alveolar injury, increased aggregation of monocytes/macrophages and neutrophils, pulmonary edema, and hyaline membrane formation, consistent with the pathology of lethal SARS in humans (24). Early use of type I IFN could ameliorate the immunopathology of SARS in mice, whereas delayed overexpression of type I IFN, aggregation of mononuclear macrophages, and dramatic increases in cytokines/chemokines are lethal factors (25). Another study in the same mouse model showed that the levels of ARDS-related cytokines were significantly increased in mice that experienced lethal lung pathological changes, suggesting that disproportionate intensity and kinetics of the host innate immune response result in severe respiratory stress and even death independent of viral kinetics (26).

Clinically, the serum levels of proinflammatory cytokines (IFN-γ, IL-1, IL-6, IL-12, and transforming growth factor β) and chemokines (CCL2, CXCL10, and IL-8) were found to be significantly higher in patients with severe SARS than in patients with nonsevere SARS (27). In addition, the levels of IL-18, IFN-γ-inducible protein-10 (IP-10), monokines induced by IFN-γ, and monocyte chemotactic protein-1 (MCP-1) were significantly higher in patients who died than in those who survived, suggesting that IFN-γ-related cytokine storms are involved in immunopathological injury in SARS patients (28). These studies showed that, in the early stage, SARS is characterized by high serum levels of proinflammatory cytokines (IL-6, IFN-α, IFN-γ) and chemokines as well as high expression levels of IFN-stimulated genes (ISGs). An uncontrolled interferon response may lead to the failure of the transition from innate immunity to acquired immunity, which is closely correlated with poor disease prognosis (28, 29).

The incidence of ARDS in COVID-19 patients is as high as 14.8–17% (6, 30) but as high as 61.1% in patients in the intensive care unit (ICU) (10). Evidence from COVID-19 patients in the ICU suggests that high serum levels of proinflammatory cytokines (IL-2, IL-7, granulocyte colony-stimulating factor, IP-10, MCP-1, MIP1A, and TNF-α) play an important role in the pathogenesis of COVID-19 (31). Patients with severe disease exhibited higher serum levels of IL-2R and IL-6 than patients with mild or moderate disease (32). Similar results were found in two other clinical studies: the levels of IL-2R, IL-6, and TNF-α were significantly higher in patients with severe COVID-19 than in those with moderate disease, suggesting that the cytokine storm is a key factor in the deterioration of patients with COVID-19 (33, 34). Additionally, patients with severe COVID-19 exhibited significant reductions in the peripheral blood lymphocyte, T lymphocyte, CD4^+^ T cell, and CD8^+^ T cell counts. These findings confirmed that SARS-CoV-2-related SIRS and ARDS exist in patients with severe COVID-19 and that acute kidney injury and myocardial injury are present in patients who die of COVID-19. Another study found that CD4^+^ T lymphocytes in COVID-19 patients are rapidly activated to become pathogenic T-helper (Th) 1 cells and produce granulocyte macrophage colony-stimulating factor and other cytokines, further inducing the generation of CD14^+^CD16^+^ monocytes that produce high levels of IL-6 and consequently accelerating the inflammatory response. Intrapulmonary accumulation of abnormally activated immune cells can induce immune damage, leading to pulmonary dysfunction and rapid death. The excessive, ineffective host immune response of pathogenic T cells and inflammatory monocytes may be associated with severe pulmonary pathology (35).

The results of the above studies suggest that the severity of COVID-19 may be attributed to the decreased function of sentinel macrophages, the excessively delayed response of DC and alveolar epithelial cells to cytokines and chemokines, the formation of an inflammatory cytokine storm, monocyte infiltration, and lymphocyte dysfunction. In the early stage of severe COVID-19, the inflammatory cytokine storm leads to mixed antagonistic response syndrome (MARS), which is dominated by SIRS and results in ARDS, coagulation dysfunction, acute renal injury, myocardial injury, multiple organ dysfunction syndrome (MODS), and even multiple organ failure (MOF). In the late stage of disease, increasing levels of anti-inflammatory cytokines (IL-4, IL-10) inhibit the activation of immune cells, and the immune response of MARS shifts to domination by compensatory anti-inflammatory response syndrome (CARS), which leads to immune exhaustion and susceptibility to secondary infections.

## Immune Evaluation and Indicators for Severe COVID-19 Pneumonia

Significant decreases in circulating T lymphocyte, CD4^+^ T cell, and CD8^+^ T cell counts, which are associated with disease severity, have been found in both SARS and COVID-19 (10, 36, 37). The absolute numbers of T lymphocytes, CD4^+^ T cells, and CD8^+^ T cells in patients with severe disease (294.0, 177.5, and 89.0 × 10^6^/L, respectively) were found to be significantly lower than those in patients with moderate disease (640.5, 381.5, and 254.0 × 10^6^/L), indicating that total lymphocyte counts and T cell subset counts can be used to evaluate disease severity (33). A study showed that patients in the early stage of COVID-19 who were ≥50 years of age and had a peripheral blood neutrophil-to-lymphocyte ratio (NLR) of ≥3.13 were likely to develop severe disease (38). Another study showed that the neutrophil count was significantly increased in COVID-19 patients not only at onset but also 13–15 days after onset, while the lymphocyte count continued to decline; in parallel, levels of both inflammatory (IL-6, IL-2) and anti-inflammatory (IL-4, IL-10) cytokines increased (39). IL-6 peaked within 3 days and then decreased briefly at 4–6 days. IL-10 peaked at 4–6 days; thereafter, the levels of IL-6 and IL-10 remained high but began to decline after 16 days. IL-6 can also be used to predict severe sepsis (40). The results of that study suggest that, in patients with severe COVID-19, SIRS may occur very soon (≤3 days), and a mixed response with CARS—i.e., MARS—subsequently begins on days 4–6. Lymphocytes were found to be gradually depleted as the disease progressed, resulting in immunosuppression (on approximately day 16), manifesting as atrophy of the immune organs, secondary infection, and MODS. A similar clinical process was confirmed in another clinical study: the median time from onset to dyspnea was 8 days in patients with severe COVID-19, and rapid progression within 3 days usually predicted a poor prognosis (31). Other studies showed elevated levels of IL-6 in COVID-19 patients, and the serum IL-6 level was significantly higher in patients with severe disease than in those without severe disease (108 ± 12 ng/L *vs* 34 ± 7 ng/L) (32–34). These findings suggest that the serum IL-6 level can be used as a key indicator of disease severity. The IL-6/IL-10 ratio may also be an important indicator for assessing the immune status of COVID-19 patients (27), although no established cutoff value is available to identify SIRS and/or MARS. Considering the aforementioned studies collectively, we believe that disease stage should be considered when serum IL-6 levels are used to evaluate the immune status of patients with COVID-19 and that the predictive value of this parameter is relatively low 16 days after disease onset. In addition, the serum IL-2R level was found to be significantly higher in patients with severe COVID-19 than in patients without severe disease (1185 ± 80 U/ml *vs* 631 ± 37 U/ml) (32, 33), suggesting that serum IL-2R may also play a role in predicting the severity and prognosis of COVID-19. Since both SARS and COVID-19 patients exhibit intrapulmonary infiltration primarily of macrophages, the serum MCP-1 level may be an alternative indicator of disease severity (7, 41). IFN-γ is a key factor causing cytokine storms (28); thus, serum IP-10 and ISG levels are suitable for the assessment of disease severity (27, 29, 31, 41). The level of the neutrophil chemokine IL-8 can also be used to assess the disease condition (41, 42).

In summary, an increase in the IL-6 level to five times the upper limit of normal; an NLR of >3.13; a peripheral blood lymphocyte count of <500 × 10^6^/L; and significantly increased serum levels of IL-2R, IL-8, IFN-γ, and IP-10 may be indicators of SIRS. Early and timely immunosuppression may attenuate or even block the development of ARDS and/or MODS caused by SIRS. On the other hand, significantly elevated IL-4 and IL-10 levels and a progressively reduced peripheral blood lymphocyte count may suggest an immune status dominated by CARS, for which immune enhancement therapy is needed.

## Immunotherapy Strategies for Patients With Critically Ill COVID-19 Pneumonia

**Figure 1:**
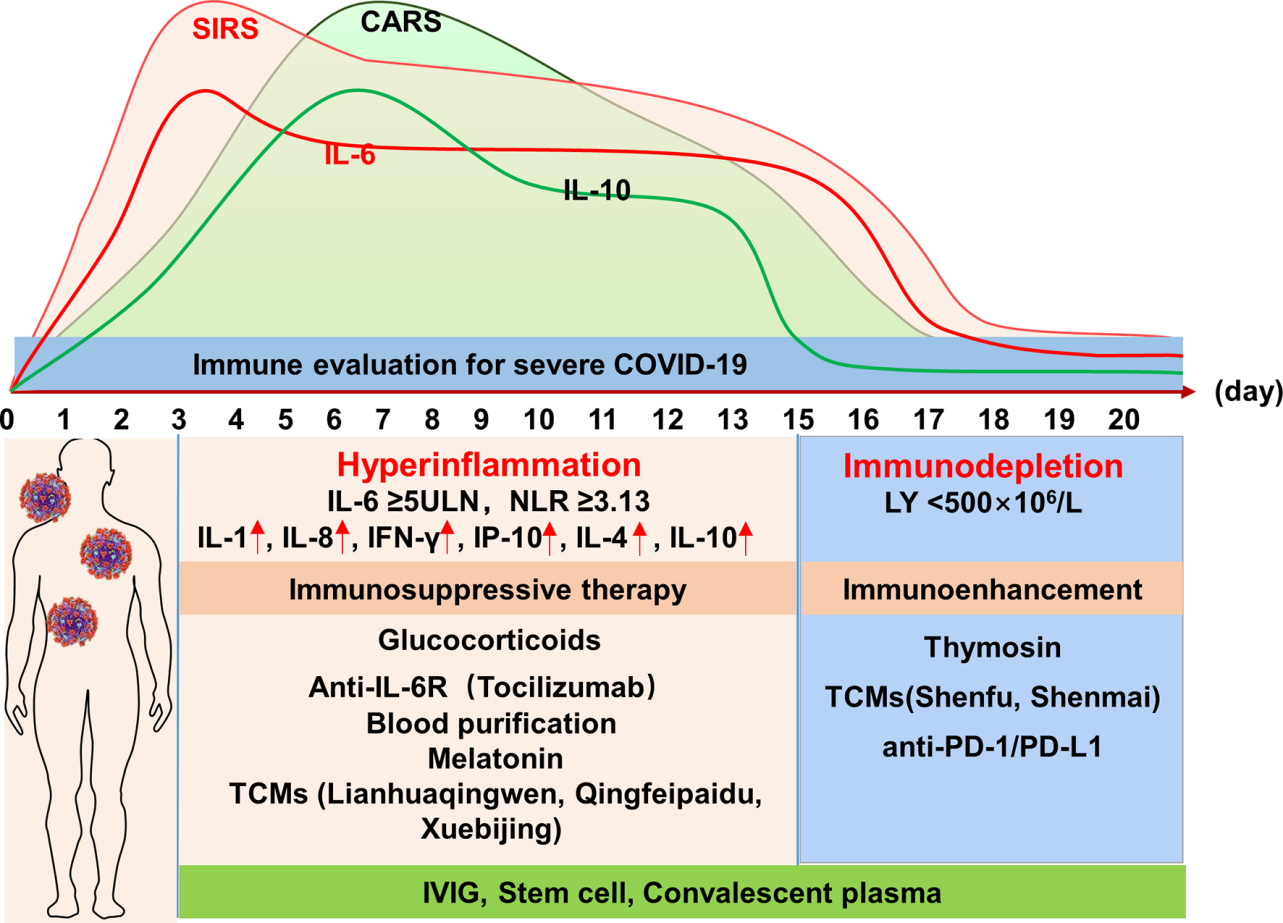
Profile of the immune response of severe COVID-19 and potential immunotherapeutic approaches. IL, interleukin; NLR, neutrophil-to-lymphocyte ratio; LY, lymphocyte; IFN-γ, interferon-γ; IP-10, IFN-γ-inducible protein-10; TCMs, traditional Chinese medicines; IVIG, intravenous immunoglobulin; PD-1, programmed cell death-1; PD-L1, programmed death ligand 1.

In addition to vaccine development and approaches that directly target the virus or block viral entry, treatments addressing the immunopathology of the infection have become a major focus. Critically ill patients with COVID-19 are immunopathologically characterized by SIRS-CARS-MARS and ARDS; SIRS predominates, leading to ARDS in the early stage and ultimately evolving to domination by CARS, resulting in the suppression of immune function in the later stage. Therefore, immunosuppressive or immune enhancing therapy should be applied as appropriate according to the disease course and as determined by monitoring the immune status (**Figure 1**).

### Immunosuppressive Therapy

Because SIRS and ARDS are characterized by cytokine storms, immunosuppressive strategies that can be adopted include glucocorticoids to suppress the intensity of the immune response, monoclonal antibodies specific for proinflammatory cytokines, blood purification to remove inflammatory mediators, traditional Chinese medicines (TCMs), and stem cell therapy.

#### Glucocorticoids

Glucocorticoids are the most common anti-inflammatory drugs; they can inhibit inflammation, and they can also inhibit the immune response and pathogen clearance, leading to potent side effects. During the SARS epidemic, excessive use of glucocorticoids was suspected to cause femoral head necrosis or diabetes. The steroid toxicity of glucocorticoids also leads to affective mental disorders in combination with personal vulnerability and possible psychosocial stressors. Moreover, early use of glucocorticoids increases the plasma viral load, with increased peak lactate dehydrogenase levels. The application of glucocorticoids in critically ill patients with COVID-19 has been controversial. Some studies have shown that the use of glucocorticoids delays the negative conversion of viral RNA in oropharyngeal swabs and feces (43) and that systemic application of glucocorticoids does not confer significant benefits (44). Excessive doses, long-term treatment, and improper selection of indications may contribute to the clinical ineffectiveness and even severe side effects of glucocorticoids. The clinical benefits of glucocorticoids may depend on the indication for glucocorticoid treatment (disease severity) and the timing, dose, and duration of intervention. In a large-sample study on SARS treatment, the crude mortality rate of the 1188 patients receiving steroid treatment was lower than that of the 99 patients in the control group (17.0% *vs* 28.3%); the patients who received low-dose oral prednisolone and those that received high-dose methylprednisolone had the lowest mortality rates (45). These findings suggest that the application of corticosteroids with high anti-inflammatory activity at the appropriate time and dose corresponding to disease severity may help to control immunopathological lung injury, thereby improving the prognosis of SARS. In another study, patients receiving the early methylprednisolone shock treatment regimen exhibited lower oxygen demand and better imaging improvement, without increased side effects, than patients receiving other corticosteroid regimens (46). In a study on COVID-19 pneumonia, patients with severe disease were administered glucocorticoid treatment (median hydrocortisone equivalent of 400.0 mg/day) for an average of 9.5 days. The blood oxygen saturation (SaO_2_) and arterial partial pressure of oxygen (PaO_2_) increased 3–5 days after treatment, and the cytokine storm was effectively inhibited in the ARDS stage, which allowed time for disease control and did not increase mortality (47). Recently, a randomized controlled clinical trial (RECOVERY trial) conducted in the UK reported for the first time that dexamethasone could reduce the mortality of patients with COVID-19. Compared with 4300 patients receiving standard care, low-to-medium dose (6 mg) dexamethasone daily for 10 consecutive days reduced the mortality rate by one-third in COVID-19 patients who were on a ventilator and by 20% for those who needed oxygen but had not been on a ventilator; no significant adverse events were found (48). Recently, WHO Rapid Evidence Appraisal for COVID-19 Therapies (REACT) Working Group published the results of a prospective meta-analysis evaluating the efficacy of corticosteroids in critically ill patients with COVID-19, which pooled data of 1703 cases of critically ill patients from 7 RCTs conducted in 12 countries. Compared with usual care or placebo, administration of systemic corticosteroids was associated with lower 28-day all-cause mortality (49). An important observation from the RECOVERY trial and the above prospective meta-analysis showed that corticosteroids provided benefit only for severely ill patients with COVID-19 immunopathologically dominated by SARS-CoV-2-associated hyper inflammation. In patients with mild COVID-19, the immune response is under the control of the immune system, which, preferable for eliminating the virus, and glucocorticoids are unnecessary and may lead to delayed virus clearance (48–50). Thus, glucocorticoid therapy is recommended for patients with severe COVID-19 at the appropriate time of the early stage (SIRS stage) of disease, with the severity-dependent dosage, and for the limited treatment period in which initial impact therapy is applicable. A regimen with short-term (3–5 days), low-dose methylprednisolone (1–2 mg/kg/day) was recommended by the Chinese guidelines for COVID-19 (51).

#### Monoclonal Antibodies Specific for Inflammatory Cytokines

IL-6 is a key factor in the inflammatory cytokine storm. Tocilizumab, a humanized anti-IL-6R antibody approved for the treatment of rheumatoid arthritis, CRS, and idiopathic multicentric Castleman disease (IMCD) (52), is recommended for the treatment of COVID-19-induced CRS (53). A study showed that tocilizumab had significant therapeutic efficacy in patients with severe COVID-19; 19 of the 20 patients with severe disease who received the treatment recovered and were discharged, and the rest of the patients recovered well (54). Experimental intravenous administration of tocilizumab for the treatment of COVID-19 was carried out in China and Italy and showed encouraging results (55). In addition, a clinical trial (ChiCTR2000029765) of tocilizumab for the treatment of COVID-19 is currently underway. Other monoclonal antibodies specific for proinflammatory cytokines also show prospects for treating COVID-19, which include Canakinumab, a monoclonal antibody targeting IL-1β (56); Eculizumab, C5-targeting monoclonal antibody (57); Narsoplimab, a monoclonal antibody against mannose-binding protein-associated serine protease 2 (58); and Dupilumab, a monoclonal antibody of the immunoglobulin G4 subclass (59).

#### Blood Purification

Blood purification treatment can theoretically eliminate serum inflammatory cytokines. In the treatment of H7N9-induced severe pneumonia, which is also characterized by ARDS, plasma exchange (PE) combined with continuous veno-venous hemofiltration treatment can significantly reduce serum levels of cytokines and improve outcomes (60). Several studies have shown the attractive effects of blood purification treatment in relieving cytokine storms and improving the outcomes for patients critically ill with COVID-19 (61–63). Importantly, better efficacy can be achieved with earlier application of blood purification therapies (64–66). Blood purification has been recommended by consensus of Chinese experts for the treatment of patients with severe COVID-19 (51, 67).

#### Stem Cell Therapy

Mesenchymal stem cells (MSCs) can regulate the proliferation, activation, and effector functions of all immune cells and play an immunosuppressive role in the activity and cytokine secretion of neutrophils and macrophages (68, 69). Thus, MSCs could reduce the occurrence of cytokine storm in acute-phase responses of COVID-19. Besides their powerful anti-inflammatory and immunoregulatory abilities, MSCs can also inhibit cell apoptosis and promote endogenous tissue regeneration, which will contribute to the tissue repair from SARS-CoV-2 induced lung injury. Furthermore, MSCs can release antimicrobial molecules that will benefit the clearance of SARS-CoV-2 (70). Bone marrow MSCs have shown therapeutic efficacy in patients with ALI, ARDS, asthma, chronic obstructive pulmonary disease, or idiopathic pulmonary fibrosis (71). Seven COVID-19 patients who received ACE2-expressing bone marrow MSCs showed significant improvement in lung function and symptoms within 2 days after treatment. After 3–6 days of treatment, the peripheral blood lymphocyte counts increased; C-reactive protein (CRP) levels decreased; and excessively activated immune cells that secrete cytokines, including CXCR3^+^CD4^+^ T cells, CXCR3^+^CD8^+^ T cells, and CXCR3^+^ NK cells, disappeared. Significantly lower serum TNF-α and higher serum IL-10 levels were observed in patients who received bone marrow MSC therapy than in patients in the placebo group (72). These results show that MSCs have promising therapeutic prospects for severe COVID-19. More than 10 registered clinical trials evaluating stem cell therapy for COVID-19 are currently underway, and the results of these randomized controlled trial (RCTs) are expected to further validate the effects of MSCs.

#### Other Anti-Inflammatory Treatments

Hydroxychloroquine sulfate and chloroquine can block internal TLR signaling and reduce the production of proinflammatory cytokines, and they have been widely used to treat autoimmune diseases like arthritis (73). Hydroxychloroquine can also inhibit proinflammatory Ca^2+^-activated K^+^ channels, leading to impaired inflammasome activation (74). On the other hand, chloroquine has been shown to have significant anti-SARS-CoV-2 efficacy both *in vivo* and *in vitro* (75, 76). This efficacy is especially observed in combination with azithromycin. Therefore, the dual antiviral and anti-inflammatory effects of hydroxychloroquine are worthy of further evaluation in patients with COVID-19 pneumonia. However, the application of hydroxychloroquine failed to reduce the risk of mechanical ventilation and all-cause death in hospitalized patients with COVID-19. On the contrary, hydroxychloroquine has been reportedly associated with an increased risk of death, especially when combined with macrolide (77–79).

Convalescent plasma has been used to treat critically ill patients with COVID-19 pneumonia. On the one hand, anti-SARS-CoV-2 antibodies in convalescent plasma can neutralize the virus and facilitate control of the virus to reduce related tissue damage. On the other hand, the plasma of patients in the convalescent phase also contains antibodies specific for inflammatory cytokines, which can neutralize inflammatory cytokines and play an important role in suppressing the inflammatory cytokine storm. In a pilot study, treatment with convalescent plasma achieved good clinical efficacy in five patients with severe COVID-19 and ARDS, and all recovered (80). In another study, 10 patients with severe COVID-19 received convalescent plasma therapy. The clinical symptoms were significantly relieved within 3 days and were accompanied by increases in oxygenated hemoglobin saturation and peripheral blood lymphocyte count and a decrease in CRP levels. Moreover, obvious absorption of intrapulmonary lesions was observed within 7 days. Interestingly, the viral RNA test results became negative in seven of the 10 patients who received convalescent plasma therapy, and no adverse effects occurred in any patient (81). The efficacy and safety of convalescent plasma therapy for patients with severe COVID-19 have been verified by many additional studies (82–84), including an international investigation of a convalescent plasma trial for COVID-19 infection (85).

Melatonin is a well-known anti-inflammatory and antioxidant molecule. It has protective effects on ALI/ARDS caused by viruses and other pathogens and exhibits potential efficacy against severe COVID-19 (86).

TCM has been widely used in China in patients with COVID-19 pneumonia (**Table 1**). In addition to its antiviral activity against SARS-CoV-2, Lianhuaqingwen capsule exhibits robust anti-inflammatory effects *in vitro*, leading to significant reductions in the levels of proinflammatory cytokines, including TNF-α, IL-6, CCl-2/MCP-1, and CXCL-10/I P-10 (87). Lianhuaqingwen has been shown to be effective in the treatment of COVID-19 in many clinic trials, including RCTs (87, 93). Another widely used TCM in China for COVID-19, Qingfeipaidu, a multicomponent herbal formula, has been shown to have antiviral, anti-inflammatory, and metabolic programming activities (88). Xuebijing injection, a patented TCM developed for the treatment of SARS in China, is indicated for infection-associated SIRS. The results of a meta-analysis of 16 papers, including 1335 cases of sepsis in a Chinese population, showed that, compared with ulinastatin alone, the combination of Xuebijing with ulinastatin led to a shorter mechanical ventilation time, shorter ICU stay, improved 28-day survival rate, reduced incidence of MODS and mortality, decreased procalcitonin concentration, ameliorated acute physiology and chronic health evaluation (APACHE) II score, and reduced levels of TNF-α and IL-6 (94). Xuebijing also exhibited antithrombotic activity, which could prevent vascular embolism caused by COVID-19 (91). Xuebijing has been recommended for the treatment of severe COVID-19 in China (51).

**Table 1:** The components and efficacy of TCMs used in the treatment of COVID-19.

TCMs	Composition	Functions	Reference
**Lianhuaqingwen**	Forsythiae Fructus, Lonicerae Japonicae Flos, Ephedrae Herba Praeparata cum Melle, Armeniacae Semen Amarum, Gypsum Fibrosum, Isatidis Radix, Dryopteridis Crassirhizomatis Rhizoma, Houttuyniae Herba, Pogostemonis Herba, Rhei Radix et Rhizoma, Rhodiolae Crenulatae Radix et Rhizoma, Menthae Haplocalycis Herba, and Glycyrrhizae Radix et Rhizoma	Inhibition of viral replication Affects the viral morphology Anti-inflammatory activity	(87)
**Qingfei Paidu**	Ephedrae Herba, Glycyrrhizae Radix et Rhizoma Praeparata cum Melle, Armeniacae SemenAmarum, Gypsum Fibrosum, Cinnamomi Ramulus, Alismatis Rhizoma, Polyporus, Atractylodis Macrocephalae Rhizoma, Poria, Bupleuri Radix, Scutellariae Radix, Pinelliae Rhizoma Praeparatum cum Zingibere et Alumine, Zingiberis Rhizoma Recens, Asteris Radix et Rhizoma, Farfarae Flos, Belamcandae Rhizoma, Asari Radix et Rhizoma, Dioscoreae Rhizoma, Aurantii Fructus Immaturus, Citri Reticulatae Pericarpium, and Pogostemonis Herba	Inhibition of viral replication Anti-inflammatory activity Regulation of metabolism Immune regulation	(88–90)
**Xuebijing**	Carthami Flos, Paeoniae Radix Rubra, Chuanxiong Rhizoma, Salviae Miltiorrhizae Radix and Rhizoma, and Angelicae Sinensis Radix	Anti-inflammatory activity (preventing cytokine storms) Antithrombotic activity	(91, 92),
**Shenmai**	The main ingredients are red ginseng and Ophiopogon japonicas.	Inhibition of cytokine storm Immune regulation Antiviral activity	(92)
**Shenfu**	The main ingredients are red ginseng and monkshood.	Promoting cellular immunity Improve symptoms Relieve lung inflammation/injury Antiviral activity	(92)

### Immune Enhancement Therapies

The immunopathology in the late stage of COVID-19 is dominated by CARS. Increasing levels of anti-inflammatory cytokines (mainly IL-4 and IL-10) and numbers of Treg cells inhibit the production and activation of immune cells, resulting in a sustained reduction in the numbers and functions of immune cells. Consequently, patients become susceptible to various secondary opportunistic infections that in turn exacerbate disease progression. Timely immune enhancement therapy can rectify the immune deficiency and augment the anti-infection capacity. Available agents include thymosin, human gamma globulin, and TCMs. In addition, blood purification can remove anti-inflammatory cytokines, and administration of fresh plasma can replenish antibodies, complement, and coagulation factors.

#### Thymosin

A meta-analysis of 19 RCTs suggested that thymosin-α1 may be beneficial in reducing mortality and regulating inflammation in sepsis patients (95). Animal experiments have shown that thymosin-α1 can reduce lung injury in septic rats through the Notch signaling pathway (96). ARDS caused by pneumonia after kidney transplantation is often accompanied by profound immunosuppression and high mortality, while thymosin can significantly improve patient outcomes (97). Thymosin-α1 is recommended as an immune enhancement therapy for severe COVID-19 pneumonia in China (51).

#### Human Gamma Globulin

Human gamma globulin has been shown to be effective in the treatment of SARS patients (98). It is speculated that during the pathogenesis of COVID-19, SARS-CoV-2 causes respiratory tract injury and enters the bloodstream to cause systemic damage to ACE2^+^ cells. Human gamma globulin is believed to enhance immune function through supplementation with diverse antibodies, which are active in the prevention of secondary infection and in blocking the inflammatory cytokine storm (99, 100).

Intravenous immunoglobulin (IVIG) can also help control inflammation and inhibit T cell activation through negatively regulating TCR signaling, affecting the number and function of regulatory T cells (101). Thus, with the dual immunomodulatory properties of both anti-inflammation and anti-infection activities, IVIG is suitable for severe COVID-19 for the whole course of disease.

#### TCMs

In rats with severe sepsis, Shenmai injection combined with recombinant IL-12 (rIL-12) was found to increase survival rate (102). In patients with sepsis, emodin can significantly reduce serum IL-10 levels (103); in addition, Shenfu injection has been shown to be effective and is routinely used in the treatment of septic shock in China (104). The above TCMs show immunomodulatory effects, mainly manifested as an enhanced immune response, which may be beneficial in patients in the late CARS stage of severe COVID-19 (51).

#### Immune Checkpoint Inhibitors

The immune checkpoint mechanism is an endogenous component of the immune system responsible for coordinating the physiological immune response, maintaining self-tolerance and protecting tissues from damage. High levels of programmed cell death-1 (PD-1) and T cell immunoglobulin and mucin domain-3 (Tim-3) have been found in T cells of patients with severe COVID-19 (105), and T cell depletion and dysfunction are common in severe COVID-19 patients (106, 107). Blocking of PD-1 or programmed death ligand 1 (PD-L1) can prevent T cell death, regulate cytokine production, and protect organs from dysfunction (106, 107). A recent phase Ib trial reported that, in patients with systemic sepsis, the application of anti-PD-1 monoclonal antibody nivolumab led to restoration of the counts and functions of lymphocytes without affecting the levels of IL-6, IL-8, and TNF-α (108). Therefore, immune checkpoint inhibitors combined with IL-6/IL-6R-specific antibodies are expected to be a new hope for COVID-19 patients (109, 110). It is cautious in the application of checkpoint inhibitors in patients with COVID-19 associated ARDS, since PD-1/PD-L1 antibodies may lead to over-enhanced immune responses or even cytokine storm.

## Summary and Outlook

Critically ill patients with COVID-19 pneumonia are pathophysiologically characterized by the presence of an inflammatory cytokine storm/SIRS, which leads to ARDS and septic shock that in turn can result in tissue damage (MODS/MOF) and even be life threatening. In the late stage of disease, CARS-dominated MARS inhibits the production and activation of immune cells, leading to severe defects in immune function and rendering the patient susceptible to secondary opportunistic infections, thereby accelerating disease progression and causing high mortality. Therefore, critically ill patients with COVID-19 pneumonia require precise stage-dependent immunomodulation, i.e., early immunosuppression in the SIRS/ARDS stage and immune enhancement to combat late-stage immunodepression. Thus, the SIRS/CARS/ARDS immune status should be accurately assessed with proper indicators and/or applicable models. In-depth study of the pathogenesis in critically ill patients with COVID-19 pneumonia will further verify the efficacy of these immunomodulatory therapeutics and improve the outcomes of COVID-19 pneumonia.

## Author contributions

YZ searched the literature, performed review design, and wrote the manuscript. YC revised the manuscript and designed the figure and table. YZ and YC contributed equally to the manuscript. ZM contributed to the conception and revision of the manuscript. All authors contributed to the article and approved the submitted version.

## Funding

This work was supported by the National Science and Technology Major Project (Grant No. 2018ZX10302-206 and 2018ZX10723203-005) and the Project of Hubei University of Medicine (FDFR201902 and 2020XGFYZR05).

## Conflict of Interest

The authors declare that the research was conducted in the absence of any commercial or financial relationships that could be construed as a potential conflict of interest.
